# Etiology and management of hypertension in patients with cancer

**DOI:** 10.1186/s40959-021-00101-2

**Published:** 2021-04-06

**Authors:** Turab Mohammed, Meghana Singh, John G. Tiu, Agnes S. Kim

**Affiliations:** 1 Department of Medicine, University of Connecticut School of Medicine, Farmington, CT USA; 2 Department of Medicine, Calhoun Cardiology Center, University of Connecticut School of Medicine, 263 Farmington Avenue, Farmington, CT 06030 USA

**Keywords:** Hypertension, Cardio-oncology, Cardiotoxicity, VEGF inhibitors, Tyrosine kinase inhibitors

## Abstract

The pathophysiology of hypertension and cancer are intertwined. Hypertension has been associated with an increased likelihood of developing certain cancers and with higher cancer-related mortality. Moreover, various anticancer therapies have been reported to cause new elevated blood pressure or worsening of previously well-controlled hypertension. Hypertension is a well-established risk factor for the development of cardiovascular disease, which is rapidly emerging as one of the leading causes of death and disability in patients with cancer. In this review, we discuss the relationship between hypertension and cancer and the role that hypertension plays in exacerbating the risk for anthracycline- and trastuzumab-induced cardiomyopathy. We then review the common cancer therapies that have been associated with the development of hypertension, including VEGF inhibitors, small molecule tyrosine kinase inhibitors, proteasome inhibitors, alkylating agents, glucocorticoids, and immunosuppressive agents. When available, we present strategies for blood pressure management for each drug class. Finally, we discuss blood pressure goals for patients with cancer and strategies for assessment and management. It is of utmost importance to maintain optimal blood pressure control in the oncologic patient to reduce the risk of chemotherapy-induced cardiotoxicity and to decrease the risk of long-term cardiovascular disease.

## Background

Recent advances in antineoplastic therapies have resulted in a significant improvement in overall and disease-free survival in patients with cancer. Better strategies for early detection and effective treatment have transformed cancer from a fatal disease to a chronic condition [1]**.** However, the quality of life of these patients often remains low because of toxicities that may arise from cancer therapies. Importantly, cardiovascular toxicities, if severe, may lead to the premature discontinuation of effective treatment [2]**.** Cancer therapies have been associated with cardiomyocyte damage, heart failure, ischemia, hypertension, and rhythm disturbances [1]**.** In a study by Patnaik et al., among sixty-three thousand women with breast cancer who were followed for nine years, cardiovascular disease (CVD) was the primary cause of death, followed by breast cancer. Intriguingly, of the women who died of CVD, only 25% had a documented history of CVD at the time of their cancer diagnosis [3]**.**

As with the other cardiovascular side effects of cancer drugs, hypertension is important to recognize and treat [4]. Hypertension is a well-established risk factor for heart failure and atherosclerotic cardiovascular disease (ASCVD) in the general population as well as in patients with cancer. Hypertension increases the risk of death from stroke, heart disease, and other vascular conditions. Recent data suggest that the presence of hypertension, particularly poorly-controlled hypertension, significantly increases the risk for chemotherapy-induced cardiomyopathy and heart failure [4, 5]. Effective treatment of hypertension allows patients to tolerate maximum doses of the planned chemotherapy, yielding better control of the tumor [6].

The objectives of this review are as follows: 1) to review the classes of antineoplastic drugs that can potentially cause hypertension, summarizing the incidence, mechanism, management for each class of cancer drug; 2) to discuss other potential causes of hypertension in patients with cancer aside from cancer treatment; 3) to review the role of poorly controlled hypertension in exacerbating anthracycline and trastuzumab-induced cardiomyopathy and heart failure; 4) to discuss the importance of blood pressure (BP) control in the oncologic patient; and 5) to discuss the recommended target BP range for cancer patients and survivors.

## The relationship between cancer and hypertension

Various classes of medications used for the treatment of cancer have been associated with the development of new hypertension or exacerbation of previously well-controlled hypertension. Furthermore, hypertension has been associated with an increased likelihood of developing certain cancers and with higher cancer-related mortality. The relationship between cancer and hypertension was first suggested by Dyer et al. in 1975 [7]**.** 1233 men were followed for 14 years in a prospective study, which demonstrated an association of higher systolic and diastolic blood pressures with higher cancer-related mortality [7]**.** In a large prospective pooled cohort study, hypertension, both treated and untreated, was associated with an increased likelihood of developing cancer compared with normotensive individuals. Additionally, there was a similar increased risk of cancer mortality in patients with hypertension (7–15%) compared with normotensive patients [8]**.**

Certain types of cancers have been associated with hypertension. Hypertensive men are at a higher risk of developing prostate cancer, and hypertensive women are at a higher risk of developing endometrial and breast cancers [4]. Hypertension is also a known risk factor for renal cancer [9]. In a study by Colt et al., hypertension increased the risk of renal cancer by two-fold in Caucasian and up to three-fold in African American patients. This risk increased to four times in African American patients if they were diagnosed with hypertension for more than 25 years [10].

In a prospective cohort study by Stocks et al. of approximately 577,800 adults followed for 12 years, there was an association between hypertension and cancer incidence in men and between hypertension and higher cancer mortality in both men and women. In men, the absolute 20-year risk for development of cancer increased from 13.7% in a normotensive patient to 15.6% in a hypertensive patient. This study also noted a statistically significant positive association between hypertension and mortality from cancers of oropharynx, pancreas, rectum, lung, prostate, and bladder in men, and pancreas, breast, and malignant melanoma in women [11]**.** Grossman et al. analyzed 47,000 patients and concluded that systolic hypertension was associated with an increased risk of mortality from cancer by 23% [12].

The underlying mechanism remains unclear. Animal models suggest dysregulation of apoptosis due to elevated blood pressures [8]**.** Another hypothesis is that angiotensin II, which is elevated in hypertensive patients, can stimulate the production of vascular endothelial growth factor (VEGF), which in turn augments cancer-related angiogenesis [13]. Finally, patients with hypertension may have other risk factors, such as advanced age, smoking, obesity, and sedentary lifestyle, which increase the risk for cancer development and cancer-related mortality.

## The role of hypertension in increasing the risk for anthracycline- and trastuzumab-induced cardiomyopathy

Anthracycline-based chemotherapy is well known to potentially cause irreversible damage to the heart in a dose-dependent manner [14, 15]. Recent data suggest that the presence of hypertension, particularly poorly-controlled hypertension, significantly increases the risk for chemotherapy-induced cardiomyopathy and heart failure [4, 5]. One of the earliest studies indicating this association was a retrospective analysis of 4018 patients published in 1979, which found that patients with underlying heart disease, hypertension, or both were at a higher risk for developing doxorubicin-induced heart failure [16]**.** In a retrospective study by Hershman et al., patients with diffuse large B-cell lymphoma (DLBCL) receiving doxorubicin-based chemotherapy versus other chemotherapy were analyzed. After adjusting for cardiac risk factors, doxorubicin was associated with a higher risk of heart failure. Among patients receiving doxorubicin, both hypertension and diabetes were strongly associated with the development of heart failure [17]**.** Pre-existing hypertension has been demonstrated to be an independent risk factor for the development of anthracycline cardiotoxicity and heart failure (hazard ratio = 1.8; *p* < 0.01) [18]. The patients with hypertension had a 58% higher risk of developing CHF than those without hypertension [18].

The mechanism of anthracycline-induced cardiotoxicity is postulated to be multifactorial involving the generation of reactive oxygen species, mitochondrial dysfunction, cardiomyocyte injury, and impaired repair mechanism [19–22]. Although most of the myocardial injury is thought to occur at the time of anthracycline exposure, it can also occur many years later. Long-lived hydroxy metabolites or secondary alcohol metabolites may accumulate in the cardiac myocytes as “anthracycline signatures”, which can lead to calcium channel inactivation, disruption of cellular metabolism, and redox imbalance [15]. Salvatorelli et al. have shown that even low-dose anthracycline therapy can potentially lead to heart failure through the same mechanism, and epirubicin has a better side effect profile than doxorubicin due to less generation of secondary metabolites [23].

Any previous stressors or damage to the heart can intensify anthracycline-induced cardiotoxicity [24]. Hypertension leads to increased systemic vascular resistance, which in turn leads to pressure overload. Increased left ventricular wall stress results in the release of growth factors and cytokines, which then leads to concentric remodeling and hypertrophy. Hypertension-mediated cytokine activation can also increase the rate of downstream cell apoptosis, leading to ventricular dysfunction [25]. These mechanisms together with the cytotoxic factors of anthracyclines may accelerate the development of heart failure [24]. In 2014, Szmit et al. studied patients with lymphoma treated with R-CHOP (rituximab, cyclophosphamide, doxorubicin, vincristine, prednisone). They found that patients with preexisting hypertension more frequently developed left ventricular dysfunction compared with patients without hypertension. In turn, this caused the hypertension subgroup to have more delays in subsequent treatment, more reductions of doxorubicin doses, and early discontinuation of chemotherapy [26].

Similarly, trastuzumab-associated cardiotoxicity has been demonstrated to be exacerbated by the presence of underlying hypertension. In patients with breast cancer receiving trastuzumab with or without anthracycline, the risk factors for the development of congestive heart failure are age > 65 years, diabetes, hypertension, obesity, and smoking history [27, 28]. One of the key mechanisms for myocardial injury in these patients is the alteration in nitric oxide (NO) synthesis and release from vascular endothelial cells [29]. Patients with hypertension have increased systemic vascular resistance, which increases myocardial wall stress. Among patients receiving trastuzumab therapy, inhibition of human epidermal growth factor receptor (HER2) activity in cardiomyocytes interrupts the HER2/neuregulin pathway, which is central to NO synthesis and sarcomere preservation [30]. Disruption of this pathway reduces NO bioavailability with a concomitant increase in angiotensin-II and reactive oxygen species (ROS) [31]. These processes, in addition to the preexisting myocardial stress from underlying hypertension, culminate in endothelial dysfunction, which is a well-established contributor to the development of congestive heart failure.

Therefore, preexisting hypertension may worsen the overall prognosis of patients undergoing anthracycline-based chemotherapy and/or trastuzumab. Effective treatment of hypertension allows the patients to tolerate maximum doses of the planned cancer therapy, yielding better control of the tumor [6].

## Cancer therapies with the potential to cause hypertension

Various classes of medications used for the treatment of cancer have been associated with the development of new hypertension or exacerbation of previously well-controlled hypertension (Table 1). In this section, we discuss the classes of antineoplastic and other drug therapies commonly used in cancer treatment that can potentially cause hypertension. For each drug class, we summarize the incidence, mechanism, and management from the available literature (Table 2).

**Table 1:** List of anticancer drugs commonly associated with hypertension

VEGF inhibitors	TKI	Proteosome inhibitors	Alkylating agents	Miscellaneous
Bevacizumab	Sorafenib	Bortezomib	Cyclophosphamide	Steroids
Ramucirumab	Sunitinib	Carfilzomib	Ifosfamide	Abiraterone
Aflibercept	Lenvatinib	Ixazomib	Busulfan	Recombinant human erythropoietin
	Axitinib		Cisplatin	Cyclosporin
	Ibrutinib			Tacrolimus
	Cabozantinib			Paclitaxel
	Pazopanib			Copanlisib
	Nintedanib			Daratumumab
	Regorafinib			Elotuzumab

*VEGF* Vascular endothelial growth factor, *TKI* Tyrosine kinase inhibitor

**Table 2:** Mechanism and incidence of hypertension associated with cancer drug class and proposed treatment recommendation

Cancer Drug Class	Mechanism of Hypertension	Incidence of Hypertension	Recommended Treatment
VEGF inhibitors	endothelial dysfunction, decrease in nitric oxide and prostacyclin I, increase in endothelin, vascular remodeling, capillary rarefaction, decreased renal excretion of sodium	All Grade: 17–80% Grade 3–4: 6–9%	CCB (e.g., amlodipine) ACEI (e.g., lisinopril)
TKI	decrease in NOS activity, activation of RAAS	All Grade: 17–47% Grade 3–4: 4–6%	ACEI CCB
Proteasome inhibitors	angiotensin-induced hypertension, aortic vascular remodeling	All Grade: 3–15%	ACEI or ARB (e.g., losartan)
Alkylating agents	oxidative damage to endothelial cells, increased intimal thickness, abnormal vascular remodeling, sodium retention	All Grade: 36–50%	ACEI or ARB
Steroids	promoting sodium and water retention, intrinsic vasoconstricting properties, enhanced sensitivity to endogenous vasopressors	All Grade: up to 13%^a^	Diuretics (e.g., hydrochlorothiazide) Mineralocorticoid antagonists (e.g., spironolactone)
Calcineurin inhibitors and other immunosuppressive agents	sympathetic overactivity, increased renal sodium reabsorption (distal tubule ENaC activation), decrease in NO production, RAAS activation, altered renal PG synthesis	All Grade: 30–80%	CCB Thiazide diuretics (especially for Tacrolimus)
Taxanes	endothelial dysfunction, enhanced toxicity of bevacizumab and anthracyclines	NA	ACEI or ARB CCB
Abiraterone	increase in steroid precursors with mineralocorticoid properties (sodium and fluid retention)	NA	Mineralocorticoid antagonists Diuretics
Recombinant human erythropoietin	increased blood viscosity, direct vasoconstricting properties, increased sensitivity to endogenous vasopressors	All Grade: 30–35%	CCB

^a^Data on the incidence of steroid-induced hypertension comes mainly from pediatric population treated for acute lymphocytic leukemia (ALL)

*VEGF* vascular endothelial growth factor, *TKI* tyrosine kinase inhibitor, *NOS* nitric oxide synthase, *RAAS* renin-angiotensin-aldosterone system, *ENaC* epithelial sodium channel, *PG* prostaglandin, *CCB* calcium channel blockers, *ACEI* angiotensin converting enzyme inhibitors, *ARB* angiotensin II receptor blocker, *NA* not available

### VEGF signaling pathway (VSP) inhibitors

Angiogenesis is one of the central pathophysiological mechanisms involved in the growth and spread of tumors [32]. Vascular endothelial growth factor (VEGF), which is found in endothelial cells, fibroblasts, renal epithelial cells, and tumors, is among the most important mediators of angiogenesis [33]. VEGF binding to VEGF receptors (VEGFR) activates multiple intracellular downstream signaling pathways, including phosphoinositide 3-kinase/AKT, endothelial nitric oxide synthase (eNOS), and prostacyclin. These pathways are important for vasodilation and maintaining the integrity of the vasculature through endothelial cell survival, proliferation, and permeability [34].

VSP inhibitors are classified into three categories based on their site of action on the VEGF pathway: 1) VEGF ligand binders that prevent binding of VEGF to VEGFR [35]; 2) small molecule tyrosine kinase inhibitors (TKIs) that interrupt intracellular pathways [36]; and 3) soluble decoy receptors acting as “VEGF trap” [37].

### VEGF inhibitors

The most widely used VEGF inhibitor is bevacizumab, a humanized monoclonal antibody (mAb) targeting VEGF-A, approved by the FDA in 2004. It is commonly used to treat advanced solid organ cancers, such as colon and other gastrointestinal malignancies, non-small cell lung cancer (NSCLC), renal cell cancer (RCC), and gynecologic malignancies, among others. Other drugs in this class include ramucirumab, mAb directed against VEGFR-2, and aflibercept, a soluble decoy receptor that binds to VEGF-A, VEGF-B, and placental growth factor, preventing them from binding and activating native VEGFR.

Hypertension is the most commonly reported cardiovascular side effect of VEGF inhibitors with incidence ranging from 17 to 80% in the literature [38]. The grades of hypertension resulting as a side effect of cancer therapy is reviewed in Table 3. In a meta-analysis of more than 21,900 patients from 72 clinical trials who were treated with bevacizumab-based therapy, all-grade hypertension was documented in 25.3% of patients, and grade 3 or 4 hypertension was noted in 8.2% [40]. In another meta-analysis of 3155 patients with non-small cell lung cancer, the incidence of all-grade hypertension was reported to be 19.55% and that of high-grade hypertension 6.95% [41]. The risk factors for high-grade hypertension were older age (> 75 years old), African-American race, higher dose of bevacizumab, drug interaction with other medications, and type of malignancy (i.e., renal tumors) [42].

**Table 3:** Grades of hypertension resulting as a side effect of cancer therapy per the NCI Common Terminology Criteria for Adverse Events (CTCAE) [39]

Grade of Hypertension	Severity
Grade 1 Mild; asymptomatic or mild symptoms; clinical or diagnostic observations only; intervention not indicated	Systolic BP 120–139 mmHg or diastolic BP 80–89 mmHg
Grade 2 Moderate; minimal, local or non-invasive intervention indicated; limiting age-appropriate instrumental ADL	Systolic BP 140–159 mmHg or diastolic BP 90–99 mmHg; Recurrent or persistent elevation (> 24 h); Symptomatic increase by > 20 mmHg (diastolic) or to > 140/90 mmHg if previously WNL; Monotherapy is indicated
Grade 3 Severe or medically significant but not immediately life-threatening; hospitalization or prolongation of hospitalization indicated; disabling; limiting self-care ADL	Systolic BP > 160 mmHg or diastolic BP > 100 mmHg; Medical intervention is indicated; Requires more intensive therapy than previously used or more than one drug
Grade 4 Life-threatening consequences; urgent intervention indicated	Life-threatening consequences (malignant hypertension, transient or permanent neurological deficit, hypertensive crisis); Urgent intervention is indicated
Grade 5 Death related to AE	Death

Adapted from Cancer Institute N. Common Terminology Criteria for Adverse Events (CTCAE) Common Terminology Criteria for Adverse Events (CTCAE) v5.0. Published Nov,2017

Although the exact mechanism of VEGF inhibitor-induced hypertension is not entirely understood, potential mechanisms have been proposed [43, 44]: oxidative stress and endothelial dysfunction [45], an imbalance between vasodilators (decrease in nitric oxide and prostacyclin I) and vasoconstrictors (increase in endothelin) [46], vascular remodeling, capillary rarefaction [47] and decreased renal excretion of sodium [48]. In addition, VEGF inhibitors can cause autonomic system toxicity and sympathetic dysregulation, resulting in hypertension [49].

Hypertension induced by bevacizumab often occurs early after treatment initiation. Controlling pre-existing hypertension is recommended before initiating therapy with bevacizumab [50]. The treating oncologist must perform a thorough history and exam and order appropriate cardiovascular workup before starting bevacizumab. Plummer et al. published a consensus statement on the management of hypertension in patients receiving bevacizumab for ovarian and cervical cancer [51]. They recommend that bevacizumab can be safely initiated in patients only if BP is < 160/100 mmHg. If office BP is > 160/100 mmHg, then home blood pressure monitoring (HBPM) is advised. If BP is > 150/95 mmHg on four consecutive days, then treatment hold should be considered, and amlodipine 5 mg may be initiated for BP control [51]. Traditionally, BP has been monitored weekly, especially in the first cycle, then at an interval of every 2–3 weeks. All patients are strongly advised to maintain BP < 140/90 mmHg. In case of discrepancies between home and clinic BP, HBPM is preferred to decide on further dosing of bevacizumab. A study by Shah et al. demonstrated that a lower dose of bevacizumab was not associated with hypertension and proteinuria [52]. In the case of high-grade hypertension necessitating additional anti-hypertensives, angiotensin converting enzyme inhibitors (ACEI) may be used [51]. These recommendations were also supported by Cameron et al., who suggested maintaining BP < 140/90 mmHg while on bevacizumab therapy with weekly HBPM and regular urinalysis for proteinuria [53].

Consistent with the recent AHA hypertension guidelines, a BP target of < 130/80 mmHg is generally recommended among patients on VEGF inhibitors [54]. Although amlodipine and ACEIs are the preferred first-line choices, adding a beta-blocker to ACEI therapy in patients with heart failure or cardiomyopathy helps improve prognosis by preventing cardiac remodeling [55]. Preclinical data have suggested that nifedipine may increase VEGF levels [56]; however, it did not reduce the antitumor activity of VEGF inhibitors. In addition, newer data suggest that nifedipine can effectively reverse BP elevation associated with VSP inhibitors. Non-dihydropyridine calcium channel blockers (CCB), such as verapamil and diltiazem, are generally avoided in these patients because they inhibit CYP3A4, which metabolizes VEGF inhibitors, leading to potentially high drug levels [57, 58].

If a patient develops hypertensive urgency or crisis while on bevacizumab, then subsequent dosing is contraindicated, and an alternative treatment regimen is recommended [6]. Bevacizumab-related hypertension often resolves when the treatment course is stopped or completed. It is important to have the patient follow up in four weeks after treatment completion to adjust or discontinue antihypertensive therapy depending on BP readings. In one study, hypertension resolved in more than 82% of patients with a median duration of 87 days after the last dose of bevacizumab [59]. Once BP normalizes, annual or biannual monitoring is usually sufficient.

Aflibercept, a VEGF trap, has also been associated with an increased incidence of all-grade hypertension in patients with RCC and requires BP management along similar lines as bevacizumab discussed above [60].

### Small molecule tyrosine kinase inhibitors

Small molecule tyrosine kinase inhibitors (TKI), such as sorafenib, sunitinib, lenvatinib, and axitinib, are used for the treatment of a variety of solid tumors, including RCC, hepatocellular carcinoma, metastatic melanoma, gastrointestinal stromal tumors (GIST), and neuroendocrine pancreatic neoplasms. Other drugs in this class include pazopanib, cabozantinib, nintedanib among others. The drugs in this class of multikinase inhibitors act by interrupting downstream intracellular VEGF signaling pathways and inhibit angiogenesis [61].

Hypertension is one of the most reported adverse events documented in 17–47% of patients receiving VSP-TKIs. The postulated mechanisms for hypertension involve decreased renal NO bioavailability via downregulation of soluble guanylate cyclase activity, inhibition of intrarenal NOS activity, activation of the renin-angiotensin-aldosterone system, and decreased fractional sodium excretion [62]. In a systematic review and meta-analysis of sorafenib-induced hypertension, the incidence of new-onset all-grade hypertension was 19.1% and high-grade hypertension was 4.3% [63]. In clinical trials, sunitinib was associated with hypertension in 17–24% [64, 65]. In an observational study on patients with GIST treated with TKIs, the incidence of new-onset or worsening hypertension was 33.3% with sorafenib and 22.7% with sunitinib. However, more patients developed grade 3 or 4 hypertension on sunitinib compared to sorafenib [66]. Similar findings were reported in a review looking at the safety profile of TKIs in patients with RCC, where it was reported that sorafenib has a better side effect profile than sunitinib. Among all TKIs, axitinib was associated with the highest rates of hypertension [67].

Several studies have demonstrated that TKI-induced hypertension may serve as a biomarker of efficacy in patients with metastatic RCC. Patients who developed the adverse events of hypertension, neutropenia, and thrombocytopenia on sunitinib had a longer progression-free survival (PFS) and overall survival (OS) [68, 69]. In another study on patients with metastatic RCC on sunitinib, patients receiving angiotensin system inhibitors for hypertension had better PFS and OS compared with patients on other anti-hypertensive medications [70]. VSP-TKIs are associated with up to a 3–8% incidence of cardiotoxicity, and the addition of ACEIs or beta-blockers for hypertension is beneficial [54]. Newer treatment options like NO donors have shown promising results for TKI-induced hypertension, and further studies are underway [71].

Hypertension associated with VSP-TKIs usually does not require treatment interruption, and concomitant anti-hypertensive therapy is often sufficient. Those patients who develop hypertensive crisis or refractory hypertension may require treatment cessation. The increase in BP induced by VSP-TKIs appears to be reversible once the drug is discontinued [72–74]. In a study of sunitinib in patients with metastatic RCC or GIST and in rats, the rise in BP was reversible after sunitinib withdrawal [75]. Whether it is safe to rechallenge a patient with a VSP-TKI once BP is better controlled remains to be answered. Rechallenge may be feasible once the underlying risk factors for vascular toxicity are screened and treated. These comorbidities include coronary artery disease, diabetes, obstructive sleep apnea, and underlying hypertension [54]. In patients with well-controlled hypertension and appropriate management of other vascular risk factors, re-introduction of VSP-TKI may be possible with close BP monitoring.

Ibrutinib is a distinct small molecule TKI which acts not via VSP but by selectively and irreversibly inhibiting Bruton tyrosine kinase (BTK) and preventing chemokine-induced adhesion and migration. Ibrutinib has also been observed to cause new-onset or worsening hypertension in 18% of patients and grade 3 or 4 hypertension in 6% of patients with CLL [76]. The mechanism of hypertension and its management strategies are yet to be clarified.

### Proteasome inhibitors

Bortezomib and second-generation proteasome inhibitor (PI), carfilzomib, are used in the treatment of multiple myeloma (MM). Both have been implicated in drug-induced hypertension with carfilzomib more often than bortezomib [77, 78]. The proposed mechanism for cardiovascular adverse effect is abnormal accumulation of ubiquitinated proteins from proteasome inhibition resulting in cellular toxicity and endothelial damage [79].

In Phase III of the ENDEAVOR trial, 16% of patients on carfilzomib and 6% on bortezomib developed hypertension [78]. Another study analyzing the safety profile of carfilzomib from four Phase II trials demonstrated an aggregated 14.3% of patients developing new-onset or worsening hypertension in patients with MM [80].

In Phase I/II clinical trials, > grade 3 hypertension was reported in 5% of all treatment-naïve patients with MM on ixazomib, a third generation PI [81]. Similar incidence (6%) was reported in patients with relapsed/refractory MM (RRMM) in a Phase III trial [82]. Long-term real-world data on the incidence and management of hypertension associated with carfilzomib and ixazomib are not yet available. Since the PIs have also been associated with cardiac dysfunction, ACEI or ARB can provide both cardio-protective and anti-hypertensive effect [83].

### Alkylating agents

Cyclophosphamide, ifosfamide, busulfan, and cisplatin are commonly used in the treatment of hematologic malignancies (lymphoma, leukemia) and solid organ malignancies (head and neck cancers and genitourinary cancers). The predominant mechanism for arterial hypertension is suspected to be oxidative damage to endothelial cells, increased intimal thickness, and abnormal vascular remodeling [84]. Other postulated mechanisms include nephrotoxicity with associated sodium retention [85]. Studies among testicular cancer survivors have shown that the use of cisplatin-based chemotherapy resulted in an increased incidence of hypertension as a late effect in up to 50% of survivors [53, 86]. No antihypertensive medication is decisively superior over others for the management of hypertension associated with alkylating agents. However, ACEIs and ARBs are preferred as first-line agents for BP control due to their reno-protective properties [87].

### Glucocorticoids

Glucocorticoids are commonly used in anticancer regimens, especially for hematologic malignancies, such as lymphoma and MM. Steroids cause new-onset or worsening hypertension by promoting sodium and water retention, exerting its intrinsic vasoconstricting properties, and increasing sensitivity to endogenous vasopressors. Glucocorticoid-induced hypertension has been reported in up to 13% of patients [88]. The mainstay of BP control for these patients is dose reduction or drug discontinuation. However, if a patient requires high-dose steroids, diuretics may be added for BP control since it targets the main mechanism of fluid retention. Mineralocorticoid antagonists and ACEIs/ARBs may also be beneficial [89].

### Non-antineoplastic immunosuppressive agents

In the context of cancer management, calcineurin inhibitors (cyclosporin and tacrolimus) are commonly used for immunosuppression post-transplant for graft vs. host disease. It is also sometimes used for autoimmune diseases associated with cancers, such as autoimmune hemolytic anemia and pure red cell aplasia. Hypertension is a common adverse effect of calcineurin inhibitors with reported incidence rate ranging from 30 to 80% [90–92]. The proposed mechanism of hypertension includes changes in sympathetic activity, increased proximal tubule sodium reabsorption, renal dysfunction with distal tubule ENaC (epithelial sodium channel) activation, decrease in NO production, RAAS activation and altered renal prostaglandin (PG) synthesis [91, 93, 94]. The National Kidney Foundation/Kidney Disease Outcomes Quality Initiative recommends maintaining BP < 130/80 mmHg in kidney transplant recipients on chronic immunosuppressive agents [95]. In a recent observational study on the efficacy of different antihypertensive medications for cyclosporine-induced hypertension among renal transplant patients, all anti-hypertensives reduced systolic BP effectively. However, beta-blockers and ACEIs were associated with increased graft failure [96].

Compared to cyclosporin, tacrolimus has been associated with a lower incidence of hypertension [97]. Low-dose amlodipine has equal efficacy as other anti-hypertensives with minimal side effects [98]. ACEIs are associated with a slight decrease in GFR, hyperkalemia, and elevation of uric acid levels when used for cyclosporin-induced hypertension [99]. Similarly, diuretics can elevate uric acid levels and precipitate acute gout. Also, diuretics can lead to hypomagnesemia, which increases the risk of arrhythmias. Thus, caution must be exercised, and close monitoring is required when prescribing loop diuretics. Since tacrolimus causes hypertension by targeting the sodium-chloride transporter in the distal convoluted tubule, thiazide diuretics are especially effective [100]. Interestingly, a recent study has shown that probiotic *Lactobacillus fermentum* also reduces the incidence of tacrolimus-induced hypertension by preventing oxidative stress, NOS uncoupling, and resultant endothelial dysfunction [101].

Mycophenolate mofetil, another immunosuppressive agent, has been associated with hypertension but to a much lesser extent compared to calcineurin inhibitors. The incidence of hypertension is thought to be dose-dependent [102], and the hypertension responds well to ARBs, especially losartan [103].

### Other antineoplastic agents

Abiraterone acetate is an oral hormonal agent used in the treatment of metastatic castration-resistant prostate cancer [104]. It inhibits CYP17, a key enzyme that catalyzes the biosynthesis of androgens, more specifically dehydroepiandrosterone (DHEA) from 17-hydroxyprognenolone [105]. As a result of CYP17 inhibition, abiraterone decreases serum levels of testosterone and other androgens. Steroid precursors can be diverted to mineralocorticoid production, resulting in fluid retention and subsequent hypertension [106]. In a meta-analysis of patients with metastatic prostate cancer on abiraterone, new-onset hypertension was significantly higher in the abiraterone group compared with placebo [107]. To prevent this mineralocorticoid toxicity, it is often prescribed with prednisone. In patients who cannot tolerate prednisone, mineralocorticoid antagonists, such as spironolactone, can be considered.

Copanlisib is a phosphoinositol-3 kinase inhibitor approved for relapsed follicular lymphoma. It has been frequently associated with hyperglycemia and hypertension. Thus, it requires pre- and post-infusion monitoring and close follow-up. Depending on the patient’s BP response, dose modification or drug discontinuation may be required [108, 109].

Taxanes are widely used for the treatment of a variety of solid tumor cancers in both adjuvant and metastatic settings. Paclitaxel is used for breast, ovarian, bladder, prostate, and esophageal cancers. Docetaxel has been approved to treat breast, lung, prostate, head and neck, and stomach cancers. The mechanism of action of taxanes is to affect microtubules, resulting in cell cycle arrest and aberrant mitosis. They have been associated with endothelial dysfunction [110, 111]. When paclitaxel is used in combination with bevacizumab for breast and lung cancer, the incidence of hypertension has been shown to increase [112, 113]. Docetaxel has rarely been associated with hypertension.

Recombinant human erythropoietin (RhuEPO) has been recommended by the American Society of Clinical Oncology (ASCO)/American Society of Hematologists (ASH) for patients with cancer and anemia after comprehensive risk-benefit assessment [114]. There has been a decrease in its use over the last decade because of its side effect profile, including systemic hypertension (reported in up to 35% of patients) and thromboembolic events [115, 116]. The possible mechanisms behind EPO-induced hypertension include increased blood viscosity, direct vasoconstricting properties, increased sensitivity to endogenous vasopressors within smooth muscle cells, and vascular remodeling [117]. CCB is a good choice for BP control but when anti-hypertensives are ineffective, EPO discontinuation is advised.

Daratumumab and elotuzumab, relatively novel monoclonal antibodies used for the treatment of RRMM, have also been associated with hypertension. Grade 3 hypertension was reported in up to 6.6% of patients on daratumumab during the CASTOR Phase III trial [118]. The incidence of all-grade and ≥ grade 3 hypertension increased when daratumumab was used in combination with carfilzomib [119]. Elotuzumab used in combination with lenalidomide/dexamethasone was associated with only 1.3% of ≥ grade 3 hypertension in the ELOQUENT-2 trial [120].

## Blood pressure assessment in patients with cancer

BP assessment is usually performed in the outpatient office setting. In-clinic measurements may be subject to error due to a variety of factors including individual technique, equipment, measurement method, and time constraints. Office BP measurements can be affected by important confounders, such as pain- or anxiety-driven sympathetic overactivity and temporary medications, such as NSAIDs or steroids. Studies have shown that patients with cancer may have a higher prevalence of both white coat hypertension and masked hypertension [121]. As such, it is crucial to incorporate out-of-clinic BP measurements, which include ambulatory and HBPM, in the management of the oncologic patient.

## Target blood pressure goal in patients with cancer

Patients with cancer have traditionally been excluded in the large-scale hypertension trials. Based on the best available evidence, the 2017 ACC/AHA Hypertension guidelines outline a series of recommendations that are generally applied to the care of the oncologic patient [122].

**Fig. 1:**
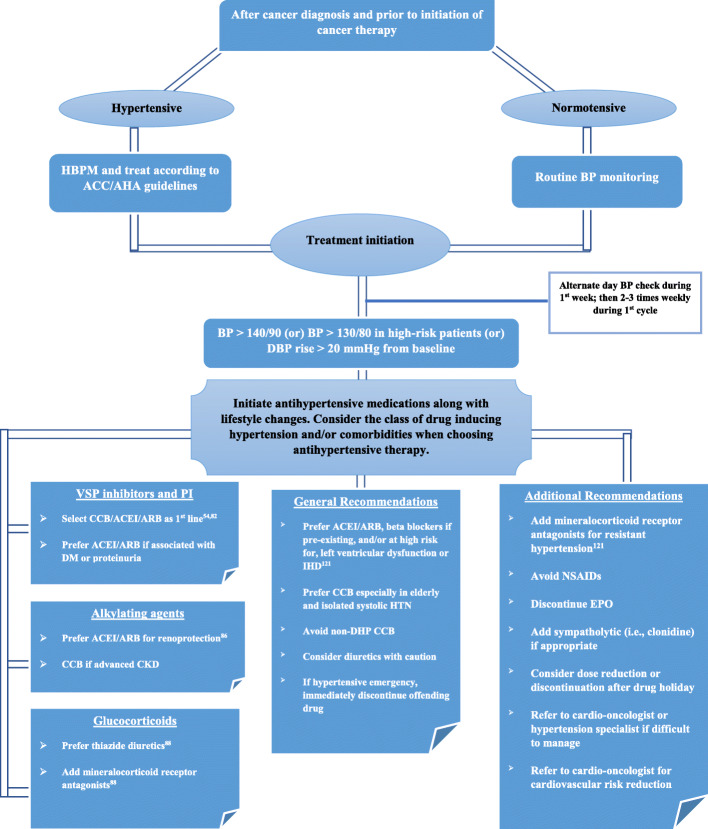
Algorithm for blood pressure assessment and management in patients newly diagnosed with cancer. BP, blood pressure; HBPM, home blood pressure monitoring; VSP, VEGF signaling pathway; PI, proteasome inhibitor; CCB, calcium channel blockers; DHP, dihydropyridine; ACEI, angiotensin converting enzyme inhibitors; ARB, angiotensin II receptor blocker; CKD, chronic kidney disease; IHD, ischemic heart disease; NSAID, non-steroidal anti-inflammatory drug; EPO, erythropoietin; DBP, diastolic blood pressure

Patients who have an estimated 10-year ASCVD risk of ≥10%, or who have additional cardiovascular comorbidities such as type 2 diabetes mellitus or chronic kidney disease, should be treated to a goal BP < 130/80 mmHg. Patients who have an estimated 10-year ASCVD risk of ≤10% with no additional cardiovascular comorbidities should be treated to a goal BP < 140/90 mmHg, though it may be reasonable to treat these patients to a goal BP < 130/80 mmHg as well [122]. Figure 1 illustrates a reasonable stepwise approach to identify and manage hypertension in the context of cancer treatment with the overarching goal of reducing the burden of CVD in this at-risk population. Randomized clinical trials with a focus on optimal BP goals and management in oncologic patients are needed to better inform contemporary clinical practice.

## Conclusion

Many studies have highlighted that the overall survival in patients with cancer is as much dependent on their comorbidities as it is on the stage of cancer at the time of diagnosis [123–126]. Cancer-related therapies are known to cause secondary hypertension as a side effect. New onset or worsening hypertension is a well-known potential adverse effect of VEGF inhibitors as well as other antineoplastic therapies. Indeed, elevated BP can be used as a surrogate biomarker for the optimal anti-tumor effect of VEGF inhibitors [68, 69]. Importantly, the treatment of resultant hypertension does not compromise the outcome of cancer treatment [127]. For patients without other cardiovascular risk factors, BP goal is < 140/90 mmHg; whereas for patients who are at high cardiovascular risk, BP goal < 130/80 mmHg should be achieved.

Given the rapid development of new treatment regimens that counter the growth and spread of cancer and increase the longevity of patients, there is an urgent need to tackle non-cancer-related comorbid medical conditions, such as hypertension, that may interfere with successful cancer treatment. Management of underlying cancer and non-cancer comorbidities must go hand in hand, and the joint efforts of the oncologist, cardio-oncologist, and primary care provider are critical to provide optimal care in these patients. The composite goal is to reduce cardiovascular events while achieving maximum benefits from cancer therapy. Timely screening for hypertension, early diagnosis and prompt initiation of intervention, regular home BP monitoring, and close follow-up can reduce the burden of cardiovascular complications, leading to an improvement in the quality of life and overall survival in patients with cancer.

## Data Availability

Not Applicable.

## Abbreviations

CVD: Cardiovascular disease
BP: Blood pressure
HBPM: Home blood pressure monitoring
VSP: VEGF signaling pathway
VEGF: Vascular endothelial growth factor
VEGFR: Vascular endothelial growth factor receptor
FDA: Food and Drug Administration
DLBCL: Diffuse large B-cell lymphoma
ROS: Reactive oxygen species
NO: Nitric oxide
HER2: Human epidermal growth factor
NSCLC: Non-small cell lung cancer
RCC: Renal cell cancer
ACC: American College of Cardiology
AHA: American Heart Association
ACEI: Angiotensin converting enzyme inhibitors
DHP: Dihydropyridine
CYP3A4: Cytochrome P450 3A4
VSP-TKI: VEGF signaling pathway tyrosine kinase inhibitors
GIST: Gastrointestinal stromal tumors
PS: Progression-free survival
OS: Overall survival
BTK: Bruton tyrosine kinase
CLL: Chronic lymphocytic leukemia
MM: Multiple myeloma
ENaC: Epithelial sodium channel
ARB: Angiotensin II receptor blocker
RAAS: Renin angiotensin aldosterone system
GFR: Glomerular filtration rate
RhuEPO: Recombinant human erythropoietin
CCB: Calcium channel blocker
ASCVD: Atherosclerotic cardiovascular disease
NSAID: Non-steroidal anti-inflammatory drug
